# Do circulating tumor cells play a role in coagulation and thrombosis?

**DOI:** 10.3389/fonc.2012.00115

**Published:** 2012-09-10

**Authors:** Garth W. Tormoen, Kristina M. Haley, Ross L. Levine, Owen J. T. McCarty

**Affiliations:** ^1^Department of Biomedical Engineering, School of Medicine, Oregon Health & Science UniversityPortland, OR, USA; ^2^Division of Pediatric Hematology/Oncology, Department of Pediatrics, Oregon Health & Science UniversityPortland, OR, USA; ^3^Human Oncology and Pathogenesis Program, Memorial Sloan–Kettering Cancer CenterNew York, NY, USA; ^4^Department of Cell & Developmental Biology, School of Medicine, Oregon Health & Science UniversityPortland, OR, USA

**Keywords:** circulating tumor cells, thrombosis, tissue factor, metastasis, blood

## Abstract

Cancer induces a hypercoagulable state, and patients with cancer who suffer a thrombotic event have a worse prognosis than those who do not. Recurrent pathologic thrombi in patients with cancer are clinically managed with anticoagulant medications; however, anticoagulant prophylaxis is not routinely prescribed owing to a complex variety of patient and diagnosis related factors. Early identification of patients at risk for cancer-associated thrombosis would allow for personalization of anticoagulant prophylaxis and likely reduce morbidity and mortality for many cancers. The environment in which a thrombosis develops in a patient with cancer is complex and unique from patients without cancer, which creates therapeutic challenges but may also provide targets for the development of clinical assays in this context. Circulating tumor cells (CTCs) may play a role in the association between cancer and thrombosis. Cancer metastasis, the leading cause of cancer-related deaths, is facilitated by the hematogenous spread of CTCs, and CTCs accompany metastatic disease across all major types of carcinomas. The role of CTCs in the pathogenesis of thrombosis has not been studied due to the previous difficulty in identifying these rare cells, but the interaction between these circulating cells and the coagulation system is an area of study that demands attention. The development of CTC detection platforms presents a new tool by which to characterize the role for CTCs in cancer-related hypercoagulability. In addition, this area of study presents a new avenue for assessing the risk of cancer-associated thrombosis and represents a potential tool for predicting which patients may benefit from anticoagulant prophylaxis. In this review, we will discuss the evidence in support of CTC induced hypercoagulability, and highlight areas where CTC-detection platforms may provide prognostic insight into the risk of developing thrombosis for patients with cancer.

## INTRODUCTION

Cancer is a hypercoagulable state, and the association of venous thrombosis with cancer has been observed for centuries (Trousseau, 1865). Thrombosis is a significant contributor to morbidity and mortality in cancer patients. Patients with cancer who suffer a thrombosis as a part of their disease endure worse outcomes (Sorensen et al., 2000). Despite the well established link between cancer and venous thrombosis, anticoagulation is not routine care for these patients (Akl et al., 2011a,b). This is due in part to the increased bleeding risk associated with anticoagulation in a population where only a fraction of patients develop symptomatic thrombosis, and the risks of anticoagulation therapy in patients treated with systemic chemotherapy and with disease and/or therapy related thrombocytopenia. No clinical marker has been developed which is capable of predicting in which patients the benefit of anticoagulation outweighs the increased risk of bleeding.

The underlying cause of thrombosis in cancer is multifactorial, including but not limited to, personal and family history of thrombosis, pre-treatment fibrinogen and platelet levels, immobility and fatigue, the increased use of indwelling catheters, and the administration of certain chemotherapeutics and other anticancer therapies which increase thrombotic risk (Dimopoulos and Eleutherakis-Papaiakovou, 2004; Elice et al., 2009). However, thrombosis can also herald the diagnosis of cancer, preceding any cancer-related symptoms or treatments. This, in itself, underlines the role for cancer in disrupting the normal physiological balance of the hemostatic system. The incidence of thrombosis in cancer is strongly tied to cancer subtype and origin, suggesting that cancer cells directly contribute to the hypercoagulable state associated with cancer (i.e., adenocarcinoma vs. carcinoma or colon vs. breast; Baron et al., 1998; Sorensen et al., 1998; Rickles and Levine, 2001; Blom et al., 2004). A multifactorial model to estimate risk of thrombosis in patients receiving chemotherapy has been validated which includes the cancer site, factors such as platelet and leukocyte count, and hemoglobin level or the use of erythropoiesis-stimulating agents, and body mass index to stratify patients by risk to develop thrombosis. However, the majority of patients predicted to have the highest risk of developing thrombosis in this model do not subsequently suffer from thrombosis (Khorana et al., 2008). Therefore, efforts to identify patients with cancer who will develop venous thrombosis need to achieve better specificity by including additional factors, such as circulating tumor cell (CTC) count, which may have a role in developing cancer-related thrombosis.

## CTC–BLOOD PROTEIN INTERACTIONS

Tissue factor (TF) is the physiological initiator of coagulation and is essential for hemostasis. TF is a transmembrane glycoprotein and requires phospholipids in order to be procoagulant (Nemerson, 1968). TF-expressing cells are not typically exposed to the blood. Only upon injury, when the architecture of the blood vessel is disrupted, does the blood gain exposure to extravascular TF-expressing cells. Therefore, in hemostasis, the activation of coagulation is essentially localized to sites of hemorrhage.

In the context of metastatic cancer, CTCs intravasate into the vasculature in order to reach distant locations and establish metastatic foci. CTCs accompany metastatic disease across all major types of carcinomas (Allard et al., 2004). Tumors have been shown to express TF *in vivo*, and TF expression has been shown to correlate with metastatic potential (Zacharski et al., 1983, 1986; Lee et al., 2011; Liu et al., 2011; Ma et al., 2011; Tian et al., 2011; Xu et al., 2011; Gil-Bernabe et al., 2012). Therefore, the process of hematogenous metastasis may present TF-expressing tumor cells to blood in the absence of a blood vessel injury. Whether metastasizing cancer cells are involved in the development of thrombosis has not been established. *In vitro*, cancer cells added to blood or plasma promote coagulation in a TF- and phosphatidylserine (PS)-dependent manner, and the coagulation kinetics are strongly dependent upon the number of cancer cells tested (Berny-Lang et al., 2011; Tormoen et al., 2011; Yates et al., 2011; Welsh et al., 2012). However, extrapolation of *in vitro* coagulation assays to *in vivo* phenomenon is not straightforward. Cancer cell lines may not reproduce CTC procoagulant phenotypes, and extrapolation of *in vitro* coagulation kinetics to physiological scenarios is complicated by dynamic physiological environments seen *in vivo*. Along these lines, blood flow is a strong determinant of procoagulant activity (Gemmell et al., 1988), yet a metastasizing cancer cell has dynamic temporal and spatial relationships with the blood which are unique from TF-bearing cells exposed at the site of a blood vessel injury. For instance, an intravasating or extravasating tumor cell is stationary relative to the blood flow, and thereby would experience rapid changes in coagulation kinetics due to the blood flow-mediated transport of coagulation factors to the stationary cell. In contrast, CTCs in the bloodstream experience very little relative blood flow, as these cells are transported by viscous forces within the flowing blood. The resulting coagulation kinetics for CTCs is therefore reliant upon diffusion of coagulation factors to/from the cell surface; thus the coagulation kinetics for CTCs is diffusion-limited.

The extent to which spatial and temporal relationships affect procoagulant activity of CTCs is a topic of recent investigation. In this issue, Lee et al. (2011) model the generation and coalescence of thrombin by procoagulant CTCs within the circulation and predict local thrombin concentration gradients surrounding the CTCs to have complex relationships with cell counts and distributions within the vasculature. This work suggests that procoagulant CTC counts strongly determine local thrombin concentrations, which would likely be prognostic for risk of developing thrombosis.

Cell-mediated coagulation requires the binding of coagulation factors from solution, followed by the assembly of enzyme complexes on the cell surface. This assembly is facilitated by the exposure of PS. The exposure of PS and subsequent assembly of enzyme complexes on the cell surface may be rate-limiting for the cell’s procoagulant activity. *In vitro*, the procoagulant activity of several cancer cell lines has been shown to correlate with the extent of PS exposure, more so than the relative expression of TF, supporting the notion that facilitation of enzyme complex assembly is the rate determining mechanism for cancer cell-mediated coagulation (Barrowcliffe et al., 2002; Pickering et al., 2004). Further, space limitations of the PS-regions can severely reduce coagulation kinetics, indicating a role for quantification of procoagulant surface area to assess the procoagulant activity of a cell (Haynes et al., 2012). This limitation suggests that the procoagulant phenotype of CTCs may be dependent upon the physical parameters (size and surface area) of CTCs. In this issue, Phillips et al. (2012a,b) demonstrate methods to utilize light microscopy to quantify the physical parameters of volume, mass, surface area, and density of CTCs, providing a novel technique to assess CTC heterogeneity in cancer-associated hypercoagulability.

Characterizing the role for procoagulant CTCs to initiate coagulation requires a method to functionally probe the ability of CTC to mediate coagulation. Surface expression of TF could be determined through immunofluorescent labeling methods, but these approaches do not capture the activity of TF. The ability of TF to initiate coagulation is dependent upon the cell membrane environment, specifically the exposure of PS, as well as an “active” or “decrypted” form of TF in order to facilitate coagulation. In this issue, Tormoen et al. (2012) describe an approach utilizing fluorescently labeled coagulation factors to characterize the procoagulant nature of CTCs. This approach has potential to functionally characterize the ability of CTCs to bind coagulation factors, which is crucial for their ability to facilitate coagulation. This functional labeling lends itself to current CTC detection platforms (Krivacic et al., 2004; Hsieh et al., 2006), providing a novel method with which CTC platforms can provide novel insight into cancer-related thrombosis.

## CTC–PLATELET INTERACTIONS

It has been shown that platelets, the primary mediators of hemostasis, play a key a role in mediating hematogenous metastasis (Gasic et al., 1968, 1973). *In vitro*, tumor cells bind platelets under shear stress (McCarty et al., 2000, 2002) and cause platelet aggregation, and this ability correlates with metastatic potential *in vivo* (Karpatkin et al., 1988; Amirkhosravi et al., 2003; Palumbo et al., 2005; Erpenbeck et al., 2010). The mechanisms by which platelet interactions confer metastatic potential upon CTCs are complex. Platelets can deter NK cell destruction of CTCs in the blood (Karpatkin et al., 1988; Im et al., 2004; Kopp et al., 2009). Releasates from activated platelets include growth factors such as platelet-derived growth factor, vascular endothelial growth factor, and transforming growth factor beta, and may support tumor growth and may promote the establishment of metastatic tumor sites (Kepner and Lipton, 1981; Assoian et al., 1983; Mohle et al., 1997; Nierodzik and Karpatkin, 2006). Recently, a murine model of superficial venous thrombophlebitis, a specific clinical presentation of thrombosis that can be associated with mucinous tumors, was shown to depend upon platelet activation (Shao et al., 2011). However, the role for platelet–CTC interactions for venous thrombosis has not been established in different cancer-related contexts.

## LEUKEMIA CELL–BLOOD PROTEIN INTERACTIONS

While the extreme rarity of CTCs in human solid tumors has prevented their characterization, leukemias, which are composed of circulating cancer cells, or “liquid tumors,” supply vast numbers of cancer cells to the bloodstream, making their isolation straightforward. Thrombosis is commonly seen with specific leukemia subtypes, and surface expression of TF has been identified on some leukemic cells associated with coagulopathy (De Stefano et al., 2005; Falanga et al., 2008; Liu et al., 2008; Falanga and Marchetti, 2009; Ku et al., 2009; Musil and Krc, 2010). However, converse to solid tumors and certain leukemia subtypes, hemorrhage is more common in most acute leukemias as compared to thrombosis (Barbui et al., 1998; Barbui and Falanga, 2001; Falanga and Barbui, 2001; Falanga and Rickles, 2003). Suggested mechanisms by which leukemias induce hemorrhage include bone marrow crowding and suppression of platelet production leading to thrombocytopenia. Acute myelogenous leukemia French-American-British subtype M3 or acute promyelocytic leukemia (APL), is known to surface-express TF as well as Annexin II (Menell et al., 1999), and is associated with the highest risk of thrombosis and bleeding amongst all leukemia subtypes. TF activity has been demonstrated from APL cells *in vitro*, suggesting that these cells are procoagulant. However, rather than developing focal thromboses, patients with APL present with a diffuse coagulopathy that consumes the coagulation factors within the blood leaving the patient unable to maintain hemostasis, resulting in a hemorrhagic phenotype. The surface expression of Annexin II has been shown to correlate with incidence of hemorrhage, and an Annexin II-mediated binding of tissue type plasminogen activator has been demonstrated *in vitro* (Menell et al., 1999). Therefore, in certain conditions, leukemia cells may initiate and propagate coagulation, while simultaneously facilitating anticoagulation, with the net coagulant effect resulting in consumption of coagulation factors until the patient is severely deficient and deposed toward clinically severe hemorrhage.

## SUMMARY

Metastasizing CTCs must survive the blood circulation, and the role that blood protein and blood cell interactions with CTCs plays in the progression of metastatic disease in human cancers remains ill-defined. The association between cancer and thrombosis suggests that the circulation is not inert toward CTCs, however the characterization of human CTC and blood interactions has been hampered by the technical difficulty in isolating and analyzing these rare cells. The emergence of clinical CTC detection platforms in recent years has opened the possibility to characterize the role that CTCs play in metastatic disease. One of the challenges facing researchers in this field is how to functionally characterize CTCs within the technical constraints that current CTC detection platforms require. This special edition of Frontiers in Oncology describes several methods with which CTCs are biophysically characterized using light microscopy techniques (see Phillips et al., 2012a,b). The development of CTC detection platforms to probe procoagulant properties of these cells presents new opportunities for investigators to understand the relationship between CTCs and blood proteins and/or blood cells. This theme is explored in Tormoen et al. (2012). Finally, understanding the complex environment that CTCs within the blood circulation traverse, and the effects that this environment has on the procoagulant phenotype of CTCs is addressed in Lee et al. (2012). The therapeutic targeting of CTCs may provide new insight into metastatic disease, including methods to interrogate the procoagulant nature of CTCs and understand how these relationships impact cancer progression and cancer-related morbidities and the response to antithrombotic therapies. 

## Conflict of Interest Statement

The authors declare that the research was conducted in the absence of any commercial or financial relationships that could be construed as a potential conflict of interest.
